# The *CGA1*-*SNAT* regulatory module potentially contributes to cytokinin-mediated melatonin biosynthesis and drought tolerance in wheat

**DOI:** 10.1186/s12870-025-06313-3

**Published:** 2025-03-07

**Authors:** Roohollah Shamloo-Dashtpagerdi, Angelica Lindlöf, Massume Aliakbari

**Affiliations:** 1 Department of Agriculture and Natural Resources, Higher Education Center of Eghlid, Eghlid, Iran; 2 School of Bioscience, University of Skovde, Skovde, Sweden; 3 Department of Crop Production and Plant Breeding, Shiraz University, Shiraz, Iran

**Keywords:** Melatonin biosynthesis, Abiotic stress, Gene promoter, Gene regulatory network, Hormones interactions, Cytokinin signaling

## Abstract

**Background:**

Melatonin plays a pivotal role in alleviating abiotic stresses, yet its biosynthesis regulation in crops, particularly wheat, remains unclear. This study explores regulatory components of melatonin biosynthesis under drought stress using bioinformatic, physiochemical, and molecular approaches in contrasting wheat genotypes.

**Results:**

Bioinformatic analysis identified *SNAT*, a key melatonin biosynthesis gene, and 88 transcription factors (TFs) from 26 families as potential regulators. The regulatory network for *SNAT* highlighted *CYTOKININ-RESPONSIVE GATA FACTOR 1* (*CGA1*) as a significant TF. Under drought stress, contrasting wheat genotypes exhibited distinct *CGA1-SNAT* module expression, melatonin and cytokinin levels, photosynthetic activity, and oxidative damage. Cytokinin treatments regulated the *CGA1-SNAT* module, altering melatonin content, SPAD values, and chloroplast numbers, particularly in drought-susceptible genotypes.

**Conclusions:**

This study uncovers the pivotal role of the *CGA1-SNAT* module and its interaction with the cytokinin pathway in regulating melatonin biosynthesis during drought stress. These findings enhance our understanding of the molecular mechanisms underpinning drought tolerance and offer promising targets for genetic and biochemical interventions to improve crop resilience.

**Supplementary Information:**

The online version contains supplementary material available at 10.1186/s12870-025-06313-3.

## Introduction

Crop plants frequently encounter environmental stressors that profoundly affect their growth and productivity. Among these, drought is one of the most severe, with its frequency and intensity escalating due to climate change [1, 2]. Over evolutionary timescales and through targeted breeding programs, plants have developed diverse mechanisms to mitigate the effects of environmental stress. The ability to tolerate stress, including drought, is governed by precise spatial and temporal regulation of these mechanisms [3, 4]. A deeper understanding of the underlying molecular mechanisms is required to elucidate what drives stress tolerance and, hence, advance the development of crops with enhanced resilience to adverse conditions.

Plant hormones play a fundamental role in regulating stress response pathways, with melatonin (N-acetyl-5-methoxytryptamine) uniquely controlling plant growth, development, and adaptability to unfavourable environmental conditions [5–7]. Melatonin’s diverse functions in shaping stress responses, particularly as a reactive oxygen species (ROS) scavenger, have attracted significant attention [8]. The hormone not only acts as a potent antioxidant, but also significantly increases the antioxidant capacity under oxidative stress [9–11]. This unique functionality is central to its role in stress mitigation. Moreover, previous studies have shown that melatonin influences plant photosynthetic activity under stress conditions. For example, it optimizes photosynthetic efficiency in wheat under various abiotic stresses by increasing photosynthetic rates, stomatal conductance, transpiration rates, SPAD values and intercellular CO_2_ concentrations [12–14]. As a regulatory molecule, melatonin can interact synergistically or antagonistically with other hormones and regulatory components to coordinate stress response pathways. In *Malus* plants, melatonin is essential for maintaining abscisic acid (ABA) homeostasis by modulating ABA biosynthesis and catabolism genes [15]. Furthermore, melatonin influences gibberellin (GA) biosynthesis by regulating *GA-2* and *GA-3 oxidase* [16] and ethylene biosynthesis by inducing the expression of *1-aminocyclopropane-1-carboxylic acid* (*ACC*) *synthase* and *ACC oxidase* [17]. Melatonin has also been found to regulate *NAC* transcription factor genes, which are crucial for enhancing plant responses to abiotic stresses [18]. Conversely, ABA has been shown to stimulate the expression of melatonin biosynthesis-related genes in watermelon, thereby promoting melatonin biosynthesis [19]. Jasmonic acid (JA) may aid regulation of melatonin biosynthesis in wheat under drought conditions, thereby increasing stress tolerance [13].

The functionality of plant hormones is dependent on their concentration. Precisely regulating hormone biosynthesis and degradation is crucial for maintaining their concentration and ensuring proper functionality [20, 21]. For example, it has previously been reported that wheat genotypes tolerant to abiotic stress present higher endogenous melatonin levels than susceptible genotypes [13, 22]. In rice, overexpression of melatonin biosynthesis genes led to increased melatonin production as well as improved oxidative stress tolerance under saline conditions [23]. Therefore, regulating the melatonin biosynthesis pathway is imperative for controlling melatonin levels and influencing the capacity of plants to respond to abiotic stress [24].

**Fig. 1:**
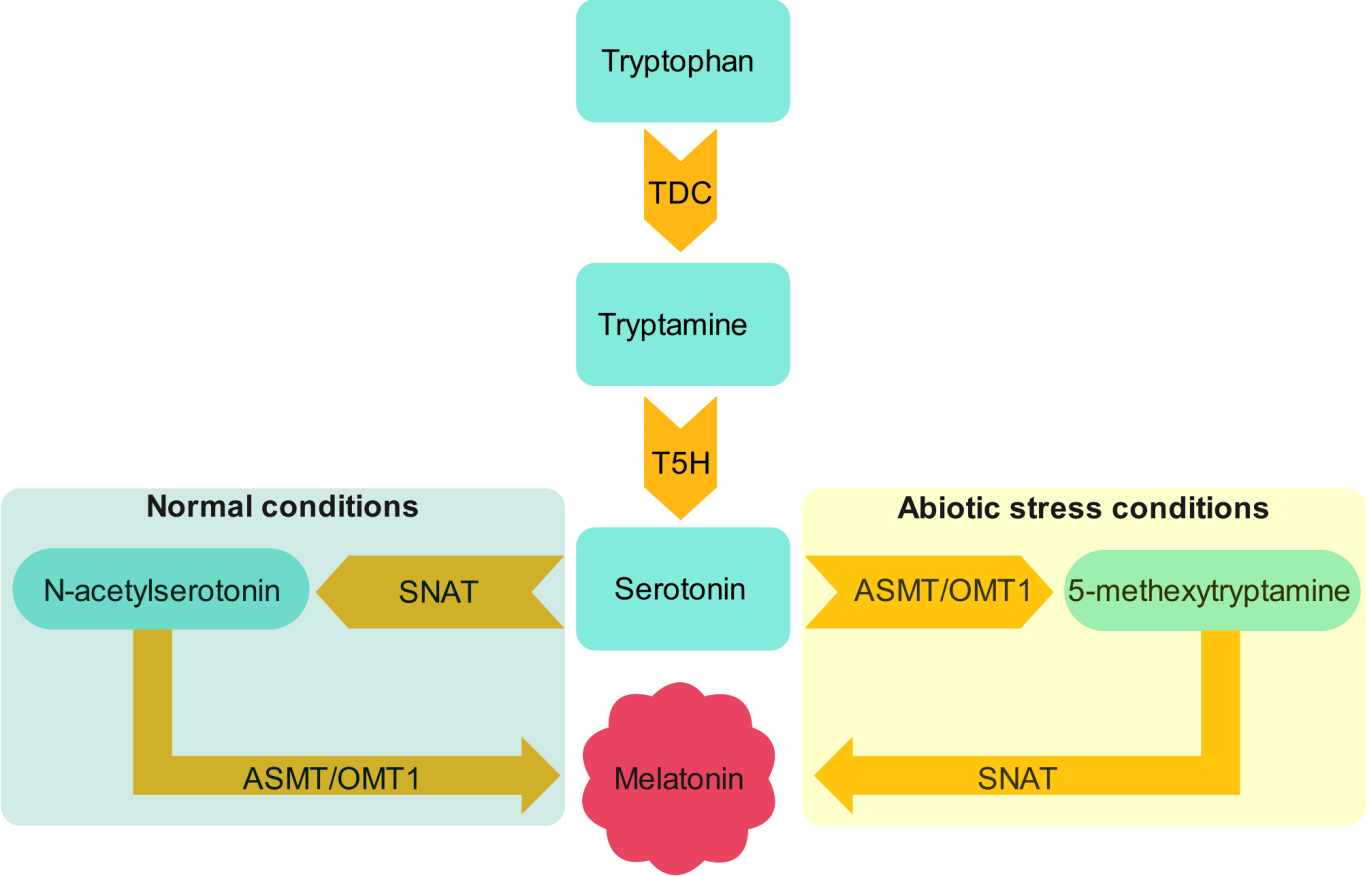
Melatonin biosynthesis in plants. Under normal conditions, tryptophan is decarboxylated to tryptamine by TDC, hydroxylated to serotonin by T5H, acetylated to N-acetylserotonin by SNAT, and methylated by ASMT/OMT1 to produce melatonin. Under stress conditions, serotonin is converted to 5-methoxytryptamine by ASMT/OMT1, and then melatonin is synthesized by SNAT

In plants, melatonin biosynthesis starts with the decarboxylation of tryptophan into tryptamine, a reaction catalyzed by tryptophan decarboxylase (TDC). The hydroxylation of tryptamine is subsequently mediated by tryptamine 5-hydroxylase (T5H), which results in the formation of serotonin. Under normal plant growth conditions, serotonin is acetylated by serotonin N-acetyltransferase (SNAT) to produce N-acetylserotonin in the chloroplast. The final step involves methylation of N-acetylserotonin, which is catalyzed by N-acetylserotonin O-methyltransferase (ASMT) or its homologues, such as O-methyltransferase 1 (OMT1), leading to the synthesis of melatonin in the cytoplasm. Under stress conditions, ASMT/ OMT1 catalyzes serotonin into 5-methexytryptamine, and then melatonin is synthesised by SNAT [25, 26] (Fig. 1). The regulation of genes involved in melatonin biosynthesis is intricate and subject to modulation by diverse environmental and endogenous factors. Under abiotic stress conditions, there is an increase in the expression of genes associated with melatonin biosynthesis, consequently leading to increased melatonin production [13, 27, 28]. Therefore, elucidating the regulatory mechanisms controlling melatonin biosynthetic genes in wheat is essential, as it can reveal new possibilities for improving drought tolerance in this indispensable cereal crop.

**Fig. 2:**
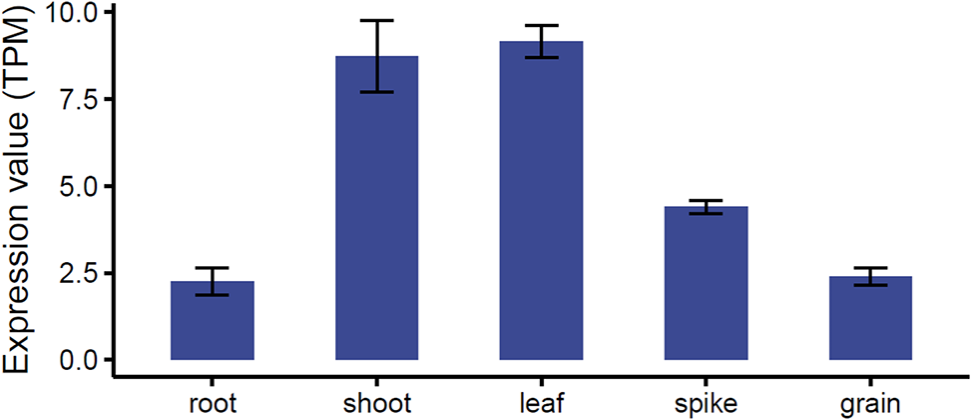
Expression profile of wheat *SNAT* gene. Expression values of the *SNAT* gene in different tissues are presented as TPM (Transcripts Per Million). Standard errors (SE) are also shown

In this study, we focused on identifying regulatory modules of *SNAT* in wheat to enhance our understanding of how its expression is regulated, particularly in response to drought stress. Bioinformatics analysis revealed a significant association between the cytokinin signalling gene *CGA1* and *SNAT*. We thereafter conducted a controlled greenhouse experiment to further explore *CGA1* and *SNAT* gene expression regulation. The study included *CGA1* and *SNAT* gene expression analysis, as well as analysis of endogenous melatonin and cytokinin levels, photosynthetic parameters, hydrogen peroxide (H_2_O_2_) concentration, and cell membrane integrity in two distinct wheat genotypes under drought conditions. Additionally, we investigated how the application of exogenous cytokinin and a cytokinin inhibitor affects the expression of *CGA1* and *SNAT* as well as the melatonin content. Our findings revealed a significant association between the regulatory module *CGA1-SNAT*, the cytokinin pathway and melatonin biosynthesis, particularly in drought stress tolerance.

## Materials and methods

### In silico analysis of wheat *SNAT* gene

The platform wGRN (https://wheat.cau.edu.cn/wGRN/) was used for retrieving gene expression profiles of *SNAT* (TraesCS1B02G312700) in wheat [29]. This resulted in expression levels generated by RNA-Seq for 407 samples from various wheat tissues. The regulator prediction module in wGRN, in combination with the hypergeometric test, significance value < 0.05 and odds ratio > 2, was used for retrieving TFs plausibly regulating the expression of *SNAT*. The GRN extraction module in wGRN was used to retrieve a gene regulatory network encompassing genes upstream and downstream of *SNAT*. Closeness and betweenness centrality analysis was conducted on the gene regulatory network in Cytoscape 3.10.0 [30, 31].

### Drought stress experiment

#### Plant material and drought treatment

Seeds from two different wheat genotypes, Sirvan (drought tolerant) and Moghan3 (drought susceptible), were used in this study [13, 32–34] and obtained from the Fars Agricultural and Natural Resources Research and Education Center, AREEO, Shiraz, Iran. Seeds of both genotypes were sterilized with 1% (v/v) sodium hypochlorite for 20 min and washed three times with deionized water, and after that, germinated and cultured with water in Petri dishes on two layers of filter paper in a temperature-controlled chamber. After 3–4 days, the uniformly germinated seeds were planted in plastic pots, with two plants in each pot, containing 1 kg of sandy-clay soil in a greenhouse at a temperature of 22 °C/18°C (day/night) and a 14-hour photoperiod. The plants were irrigated according to the soil field capacity (FC). At the five-leaf growth stage, the pots were divided into non-stressed and stressed pots. The non-stressed pots were kept humid (up to 80% FC) throughout the experiment. Plants were then subjected to drought stress by withholding irrigation until the FC was reduced to approximately 40%. Stressed plants were kept at 40% FC throughout the experiment by weighing the pots daily. Leaf samples were taken from non-stressed and stressed plants at three time points: 6, 24, and 48 h after initiating drought stress treatment. Four biological replicates containing one plant each were sampled, frozen immediately in liquid nitrogen, and stored at -80 °C for further use.

#### Expression analyses of the wheat *CGA1-SNAT* module

Total RNA was extracted using the Column RNA isolation kit (DENAzist Asia, Iran) according to the manufacturer’s instructions. RNA quality and quantity were assessed via 2% agarose gel electrophoresis and spectrophotometry at A260/230 and A260/280 nm (NanoDrop, Technologies, Inc.). Following the manufacturer’s instructions, any potential genomic DNA contamination was removed with RNase-free DNase I (Jena Bioscience, Germany). For gene expression evaluation, 2 mg DNase-treated RNA from each sample was used to synthesize first-strand cDNA using an Easy cDNA Synthesis Kit (Pars, Iran) following the manufacturer’s instructions. Gene-specific primers were designed with the AlleleID software 7.7 (Table S1), and qPCR was performed using the Bioer thermal cycler system, a 2X SYBR Green Real-Time PCR Kit (Pars, Iran), with two technical replicates per sample. Gene expression levels were calculated with the 2^−ΔΔCt^ method [35] and with *Actin* (GenBank: AB181991.1) used as the internal control (Table S1).

#### Determination of endogenous melatonin

To quantify endogenous melatonin, leaves from wheat genotypes were extracted with 1.0 mL of a chloroform: methanol (v: v, 30:1) under precooled conditions. The extract was centrifuged at 10,000 × g for 15 min at 4 °C, and the supernatant was transferred to a fresh tube and evaporated under nitrogen gas. The dried residue was then dissolved in 1.0 mL of a methanol mixture (1:1, v/v) and filtered using a 0.22 μm nylon membrane. The residue was dissolved in a 1.0 mL methanol: water mixture (1:1, v: v) and filtered (nylon, 0.22 μm). Endogenous melatonin levels in the leaf extracts were quantified with an ELISA Kit (EK-DSM; Buhlmann Laboratories AG, Schonenbuch, Switzerland), following the manufacturer’s instructions.

#### Determination of endogenous cytokinin levels

To measure free cytokinin levels, a competitive ELISA method was employed following the protocol described by Avalbaev, et al. [36]. Leaf samples, 0.5 g, were homogenized in 80% ethanol and stored overnight at 4 °C. Aqueous remnants of ethanol extracts were used to quantify the total content of zeatin derivatives, including zeatin, its riboside, and nucleotide, which were immunoreactive and were detected using rabbit antibodies against zeatin riboside in the immunoassay.

#### Evaluation of photosynthesis parameters

Several measurements were used to evaluate the photosynthetic responses of contrasting wheat genotypes to drought stress. Chlorophyll content was measured using SPAD obtained with a portable Minolta SPAD 502 Plus chlorophyll meter (Delta T, UK) [37]. Photosynthetic rate (*A*_N_; net CO_2_ assimilation), transpiration rate (*E*), and stomatal conductance (*g*_s_) were recorded using a Li-6400 portable photosynthesis system (LI-COR Inc., Lincoln, NE, USA), following [38], with some minor modifications. Measurements taken on fully expanded leaves evaluate their photosynthesis rate from 10:00 am to 1:00 pm, maintaining an average leaf temperature of 26 °C and a photon flux density of 500 mmol m^− 2^ s^− 1^. Each photosynthetic parameter was evaluated with four replicates, i.e., four pots containing one plant per pot. In addition, intrinsic water use efficiency (WUEi) was calculated as the ratio between *A*_N_ and *g*_s_ (*A*_N_/*g*_s_; µmol CO_2_ mol^− 1^ H_2_O) [39].

#### Evaluation of hydrogen peroxide (H_2_O_2_) and malondialdehyde (MDA)

H_2_O_2_ content was measured following the method described by Gong, et al. [40]. Leaf samples were homogenized in 0.1% (w/v) trichloroacetic acid (TCA) and centrifuged at 12,000 × g for 15 min. After that, 0.5 ml of 10 mM potassium phosphate buffer (pH 7.0) and 1 ml of 1 M potassium iodide (KI) were added to 0.5 ml of the supernatant. The absorbance of the mixture at 390 nm was subsequently measured using a spectrophotometer.

To evaluate drought-induced leaf tissue damage, MDA levels were quantified following the method by Heath, et al. [41]. Leaf tissue, 0.25 g, was homogenized in 5 mL of 0.1% trichloroacetic acid (TCA) and centrifuged at 3000 × g for 10 min. The supernatant was mixed with 4 mL of 0.5% TBA and heated at 95 °C for 30 min. The mixture was subsequently centrifuged again and allowed to cool to room temperature, and thereafter, MDA content was subsequently measured using a spectrophotometer.

### Cytokinin activity bioassays

#### Experimental treatments

Drought-susceptible wheat genotype Moghan3 was used for cytokinin activity bioassays. Seeds were sown and seedlings cultivated as described in Sect. “Plant material and drought treatment”. At the five-leaf growth stage, plants were divided into three groups containing four pots. The first group was treated with cytokinin by spraying with benzyl amino purine (BAP; 30 µM), the second group was treated with a cytokinin antagonist (PI-55; 10 µM) by foliar application [42, 43], and the third group (control) was sprayed with tap water.

#### Molecular, physiological, and biochemical measurements

Forty-eight hours after BAP and PI-55 treatments, several characteristics were assessed in the leaf tissues of Moghan3. Expression levels of *CGA1* and *SNAT* genes in response to experimental treatments were analysed using qPCR. Melatonin levels in leaf tissue were measured as previously described. Chlorophyll contents were measured with a portable Minolta SPAD 502 Plus chlorophyll meter. Chloroplast numbers were determined as described by Hudson, et al. [11]. Accordingly, chloroplasts were extracted from 1 g of leaf tissue using a Percoll gradient and counted with a standard 0.1 mm hemocytometer.

### Statistical analysis

Statistical analysis of molecular, physiochemical, and biochemical parameters was conducted using ANOVA and Duncan’s multiple range test, with a significant level set at *P* ≤0.05, using STATISTICA v12 software. In addition, Principal Component Analysis (PCA) based on Pearson correlation was used to determine the relationship between melatonin contents and photosynthetic parameters.

## Results

### In silico analysis of wheat *SNAT* gene

**Fig. 3:**
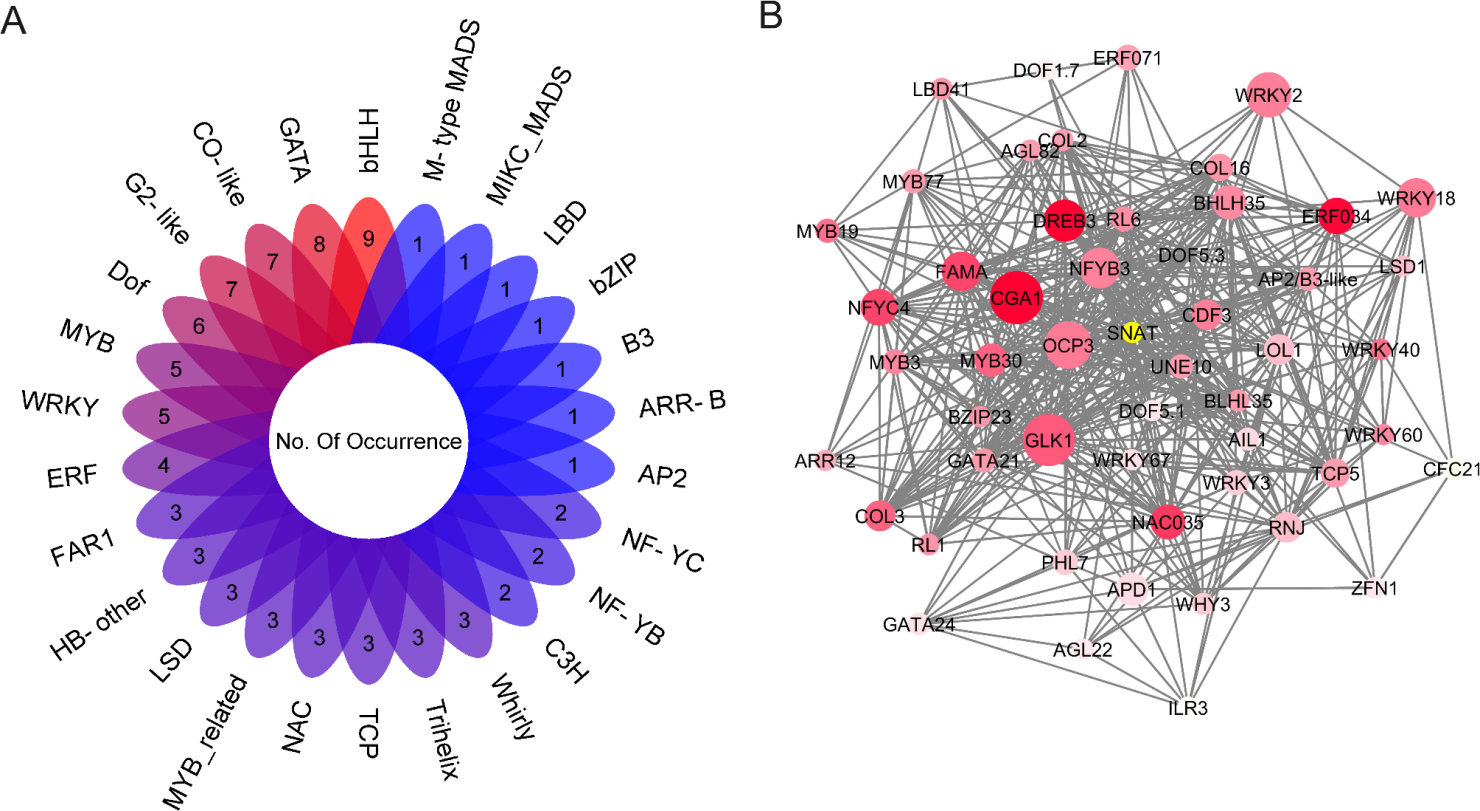
Identification of wheat *SNAT* gene regulatory modules. **A** Transcription factors families upstream of the *SNAT* gene. The number of each family member is shown. **B** Gene regulatory network of *SNAT* up and downstream genes. Nodes with larger sizes and red colors indicate a higher closeness centrality and betweenness centrality, respectively, based on the topology analysis with Cytoscape

Gene expression levels for wheat *SNAT* were quantified as Transcripts Per Million (TPM) values ± SD, for root, shoot, leaf, spike, and grain using the platform wGRN [29] (Fig. 2). Among these, the highest expression levels were observed in the leaf and shoot tissues. In contrast, root and grain exhibited the lowest expression levels. By combining the regulator prediction module in wGRN with the hypergeometric test (*P* < 0.05 and odds ratio > 2), 88 TFs from 26 different families were identified as plausibly regulators of *SNAT* expression in wheat (Fig. 3A). Notably, the bHLH, GATA, CO-like, G2-like, DOF, MYB, and WRKY families are more prominent, with > = 5 members each, compared with the other families. In contrast, the AP2, ARR-B, B3, bZIP, LBD, MIKC_MADS, and M-type MADS families are represented by only one member each.

Among the derived TFs, a GATA family member *CYTOKININ-RESPONSIVE GATA FACTOR 1* (*CGA1*-TraesCS6D02G173000) exhibited the lowest adjusted *P*-value and highest odds ratio value (Table S2). Using the GRN extraction module in wGRN, a gene regulatory network encompassing genes upstream and downstream of *SNAT* was derived and thereafter analysed to clarify if *CGA1* is a key regulator of both *SNAT* and melatonin biosynthesis in wheat. The derived regulatory network comprised 52 nodes and 1765 edges. Within this network, *CGA1* exhibited the highest closeness centrality value and a high betweenness centrality value, ranking second after *DOF5.3* (Fig. 3B). Consequently, *CGA1* was identified as a likely key regulator of *SNAT* and was selected for further analysis.

### Expression analyses of the wheat *CGA1-SNAT* module

**Fig. 4:**
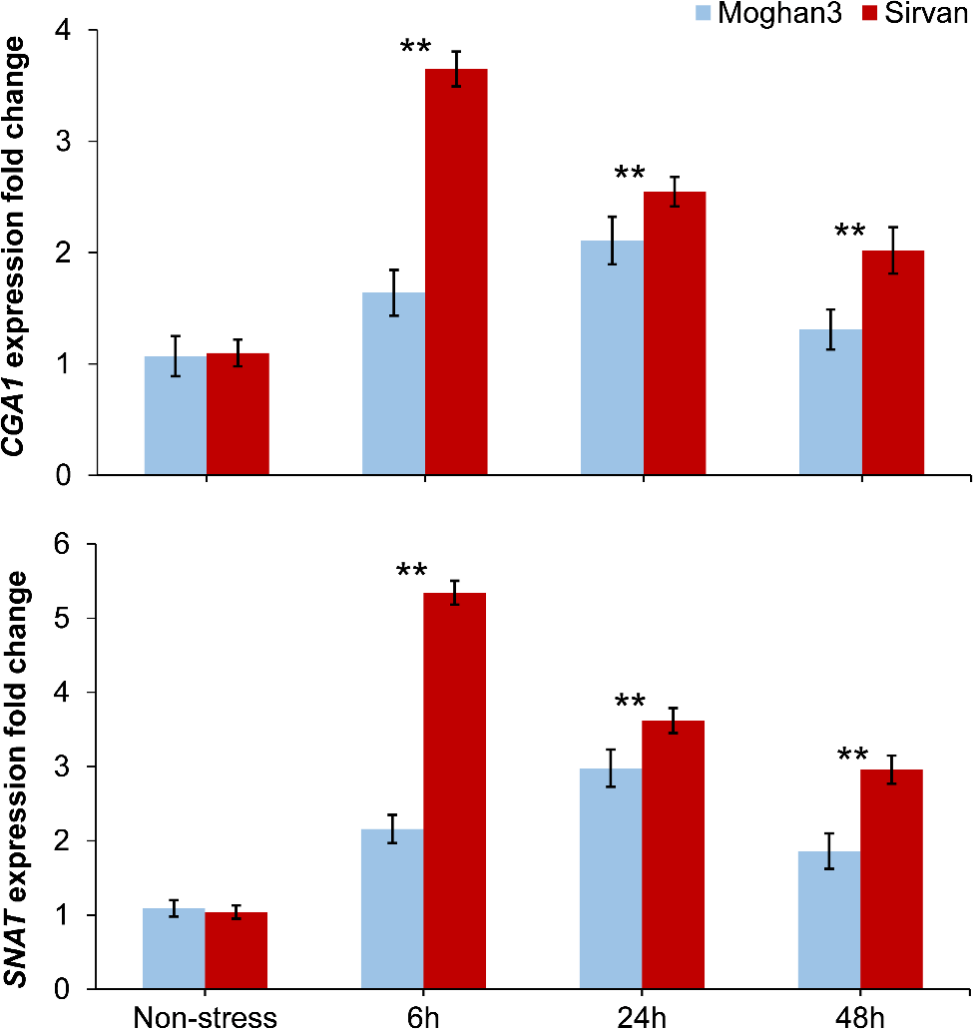
Expression profiles of the *CGA1* and *SNAT* genes in leaves of two contrasting wheat genotypes under drought conditions. The values shown are means ± SE. Asterisks indicate statistical significance based on the Duncan test (*P*-value ≤ 0.01)

The expression profiles of *CGA1* and *SNAT* genes were consistent across time points in both wheat genotypes (Fig. 4). On the other hand, the expression levels differed significantly between genotypes under the experimental conditions. Specifically, the transcript levels of both *SNAT* and *CGA1* peaked earlier in Sirvan (drought tolerant) than in Moghan3 (drought susceptible). Under drought conditions, *CGA1* expression increased up to 3.5-fold and *SNAT* expression up to 5.5-fold at 6 h time point in Sirvan. In contrast, Moghan3 showed only a 1.6-fold and 2.3-fold increase for CGA1 and SNAT, respectively, at this same time point.

Additionally, at the 24 h and 48 h time points, the expression levels were significantly higher in Sirvan compared to Moghan3 in both genotypes. Notably, there was no significant difference in *CGA1* expression at the 48-hour time point in Moghan3. Furthermore, the expression of *SNAT* was notably higher at all time points in the tolerant genotype Sirvan (Fig. 4), with maximum upregulation (up to 5-fold) of *SNAT* observed at the 6 h time point. In contrast, the expression of *CGA1* and *SNAT* peaked at 24 h after drought treatment in the susceptible genotype Moghan3.

### Determination of endogenous melatonin and cytokinin contents

**Fig. 5:**
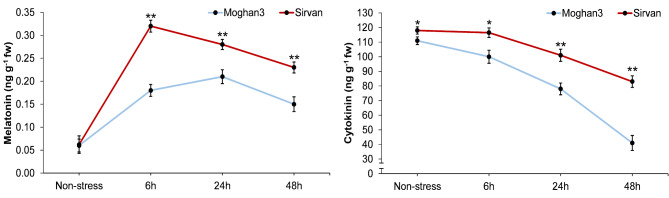
Evaluation of melatonin and cytokinin contents in leaves of two contrasting wheat genotypes under drought conditions. The values shown are means ± SE. Asterisks indicate statistical significance based on the Duncan test (*: *P*-value ≤ 0.05, **: *P*-value ≤ 0.01)

Under non-stress conditions, there was no significant difference in melatonin levels in the two wheat genotypes (Fig. 5). However, Sirvan presented significantly higher melatonin levels than Moghan3 at 6 h, 24 h, and 48 h time points. In Sirvan, the melatonin levels peaked at 6 h following drought stress, whereas in Moghan3, the peak occurred at 24 h after the onset of stress. Moreover, a difference in leaf cytokinin content was observed between the wheat genotypes (Fig. 5). In both genotypes, the highest cytokinin level was presented under non-stress conditions, and it decreased over time. However, the level was consistently higher in Sirvan than in Moghan3, even under non-stress conditions.

### Evaluation of photosynthesis parameters

Significant changes in all measured photosynthetic parameters were observed in response to drought stress in both wheat genotypes. While the SPAD value decreased significantly under drought stress in both genotypes, it remained consistently higher at all time points in the drought-tolerant genotype Sirvan compared to the susceptible genotype Moghan3 (Table 1). Additionally, both genotypes exhibited a reduction in *A*_N_, though the decrease was less pronounced in Sirvan than in Moghan3 (Table 1). Similarly, *E* and *g*_s_ declined significantly under drought stress, with Sirvan showing a greater reduction in these parameters than Moghan3 (Table 1). Despite a decline in WUE_i_ during drought, Sirvan maintained significantly higher levels than Moghan3 throughout the experiment (Table 1).

**Table 1:** Evaluation of photosynthetic parameters, including chlorophyll (Chl) content (SPAD value), photosynthetic rate as net CO_2_ assimilation (*A*_N_), transpiration rate (*E*), stomatal conductance (*g*_s_), and intrinsic water use efficiency (WUEi) of wheat genotypes under drought conditions

	SPAD value		A_*N*_ (µmol CO_2_ m^− 2^ s^− 1^)		E (mmol H_2_O m^− 2^ s^− 1^)		g_s_ (mol H_2_O m^− 2^ s^− 1^)		WUE_i_ (µmol CO_2_ mol^− 1^ H_2_O)	
	Sirvan	Moghan3	Sirvan	Moghan3	Sirvan	Moghan3	Sirvan	Moghan3	Sirvan	Moghan3
Non-stress	37.15^a^	37.27^a^	18.02^a^	17.86^a^	4.07^a^	4.10^a^	97.53^a^	97.28^a^	185^a^	184^a^
6 h	35.22^b^	35.08^c^	16.42^b^	14.38^c^	3.14^c^	3.38^b^	93.28^c^	95.03^b^	176^b^	151^c^
24 h	31.65^d^	28.61^e^	13.74^d^	11.01^e^	2.07^e^	2.66^d^	87.67^e^	91.35^d^	157^d^	121^e^
48 h	25.74^f^	20.54^g^	10.12^f^	6.89^f^	1.25^g^	2.14^f^	80.71^g^	85.33^f^	125^f^	81^g^

Different letters indicate significant differences in means based on the Duncan test (*P*-value ≤ 0.01)

**Fig. 6:**
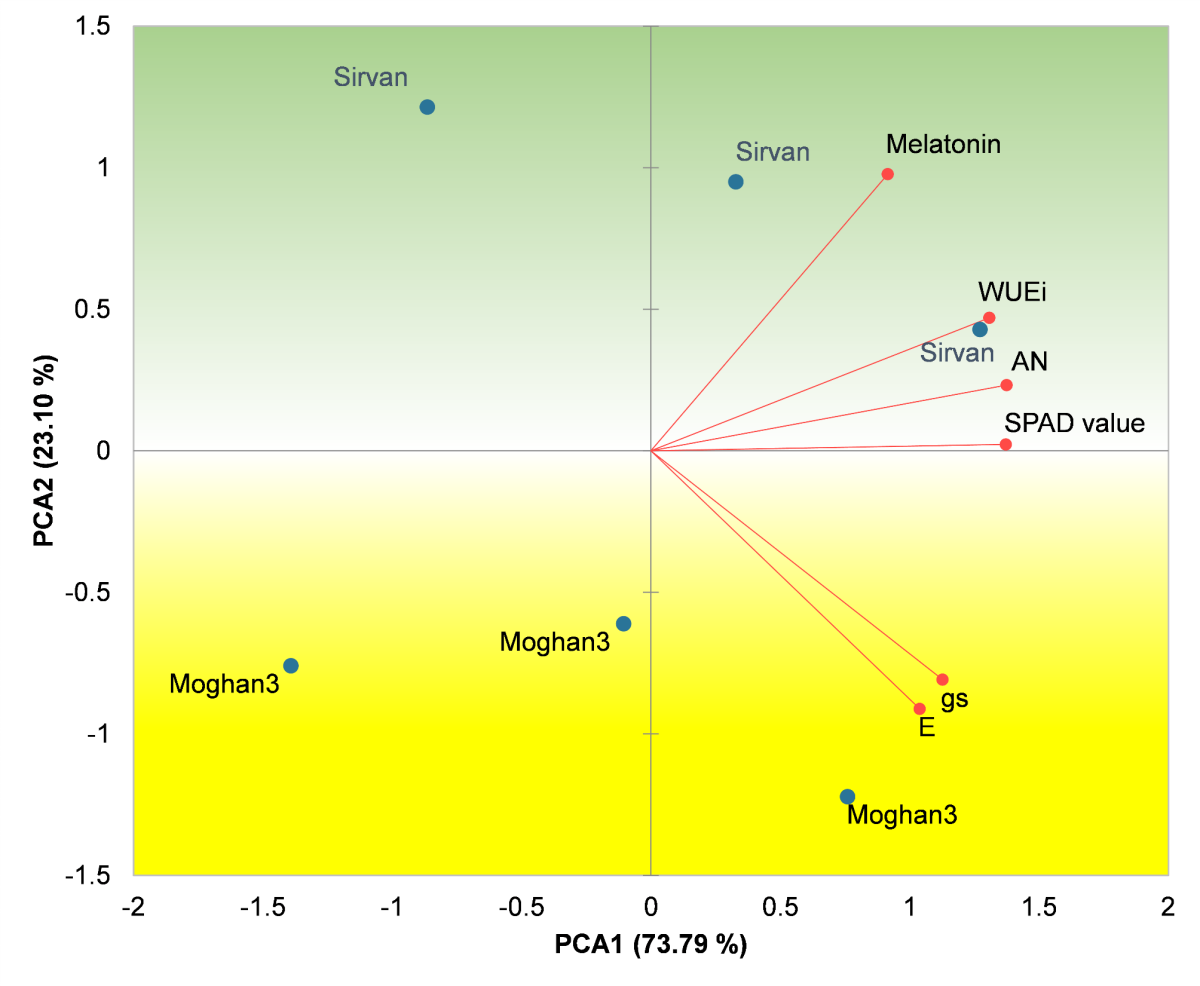
Associations among melatonin contents and measured photosynthetic parameters in leaves of two contrasting wheat genotypes under drought conditions. Green region: tolerance region (location of tolerant genotype). Yellow region: susceptibility region (location of susceptible genotype). PCA1: First principal component, PCA2: second principal component

PCA revealed a strong positive correlation between melatonin content and SPAD value, *A*_N_, and WUE_i_ (Fig. 6). In addition, PCA indicated that the drought-tolerant genotype exhibited higher melatonin content, SPAD value, *A*_N_, and WUE_i_ under experimental conditions.

### **Evaluation of H**_**2**_**O**_**2**_**and MDA contents**

**Fig. 7:**
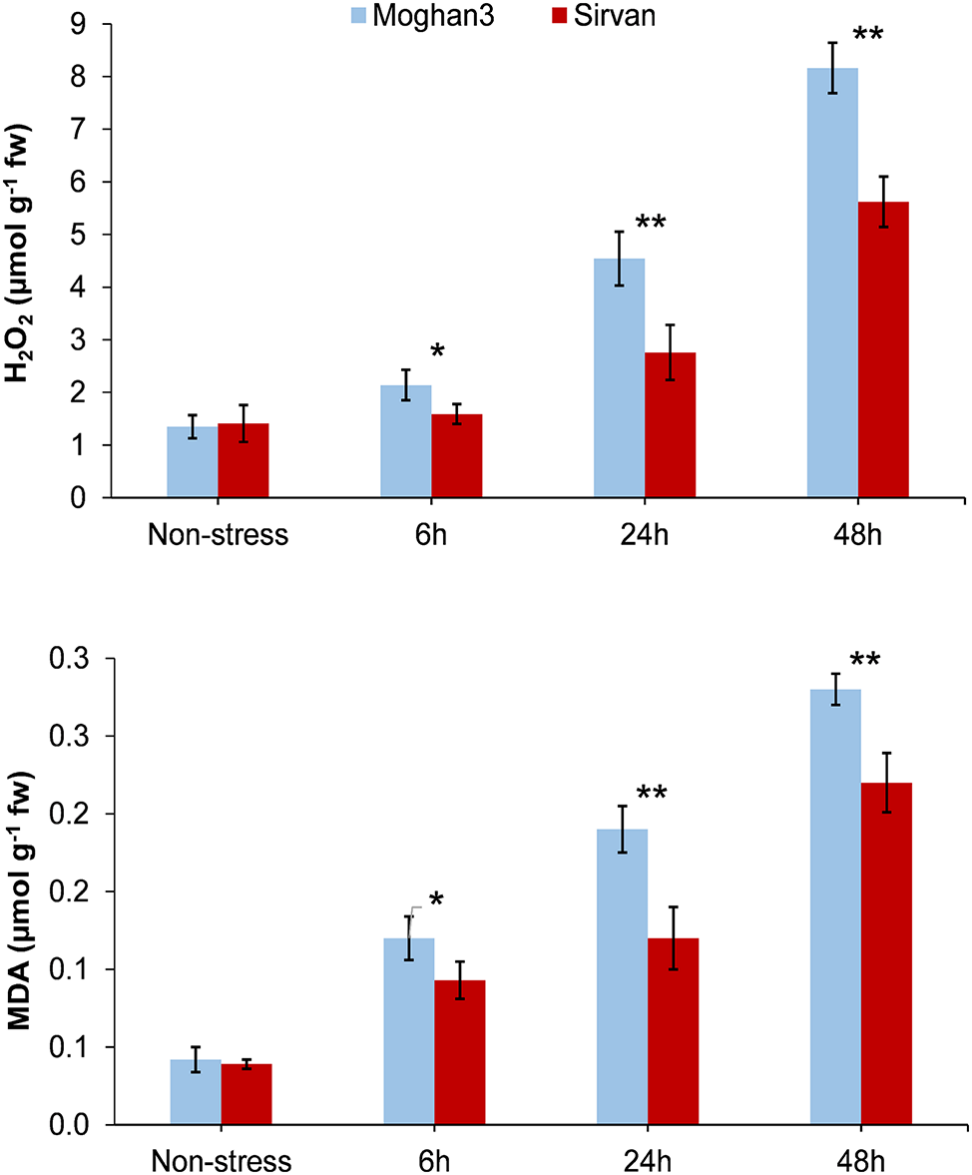
Evaluation of H_2_O_2_ and MDA levels in leaves of two contrasting wheat genotypes under drought conditions. The values shown are means ± SE. Asterisks indicate statistical significance based on the Duncan test (*: *P*-value ≤ 0.05, **: *P*-value ≤ 0.01)

Drought treatment resulted in a significant increase in H_2_O_2_ accumulation in Moghan3 at all time points (Fig. 7). However, no significant changes in H_2_O_2_ levels were observed in Sirvan 6 h after the onset of stress. Furthermore, at 24 h and 48 h, H_2_O_2_ levels were significantly higher in Moghan3 than in Sirvan. MDA also accumulated significantly in both wheat genotypes under drought stress, with higher levels in Moghan3 at all time points (Fig. 7). Additionally, the accumulation of H_2_O_2_ and MDA over time tended to be similar in both genotypes, only generally significantly higher in Moghan3 than in Sirvan.

### Effects of exogenous application of BAP and PI-55

**Fig. 8:**
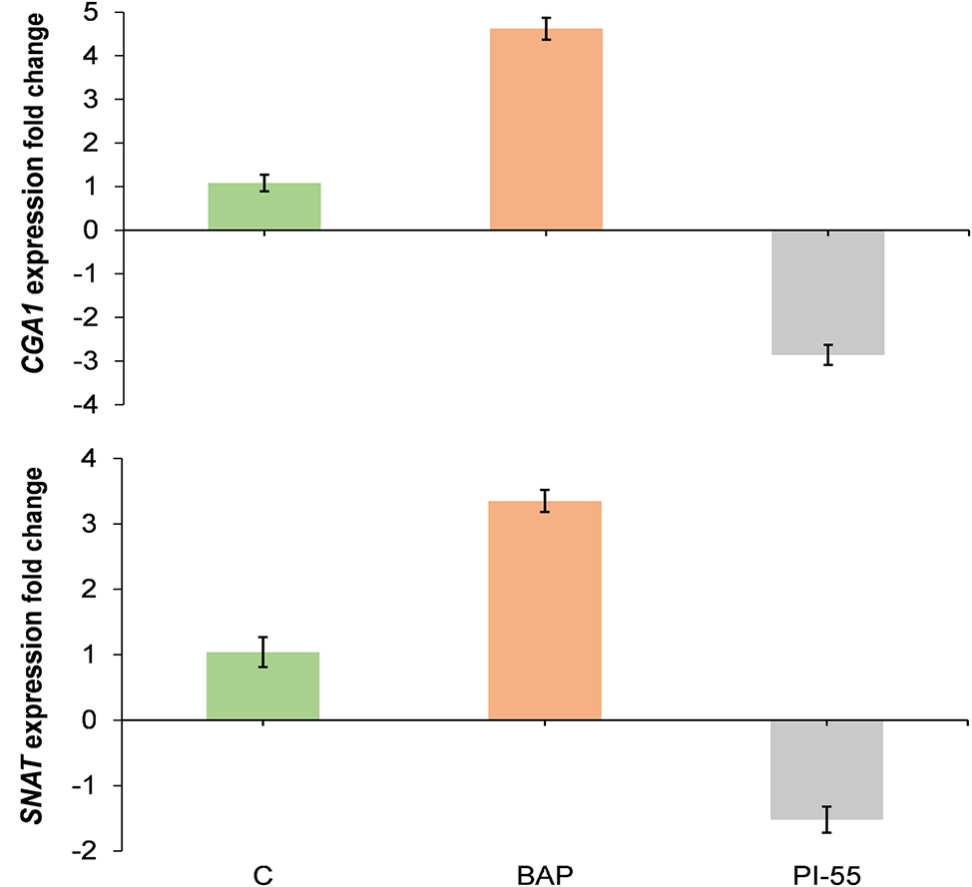
Expression analysis of the *CGA1* and *SNAT* genes in leaves of the susceptible wheat genotype treated with BAP and PI-55. The values shown are means ± SE

**Fig. 9:**
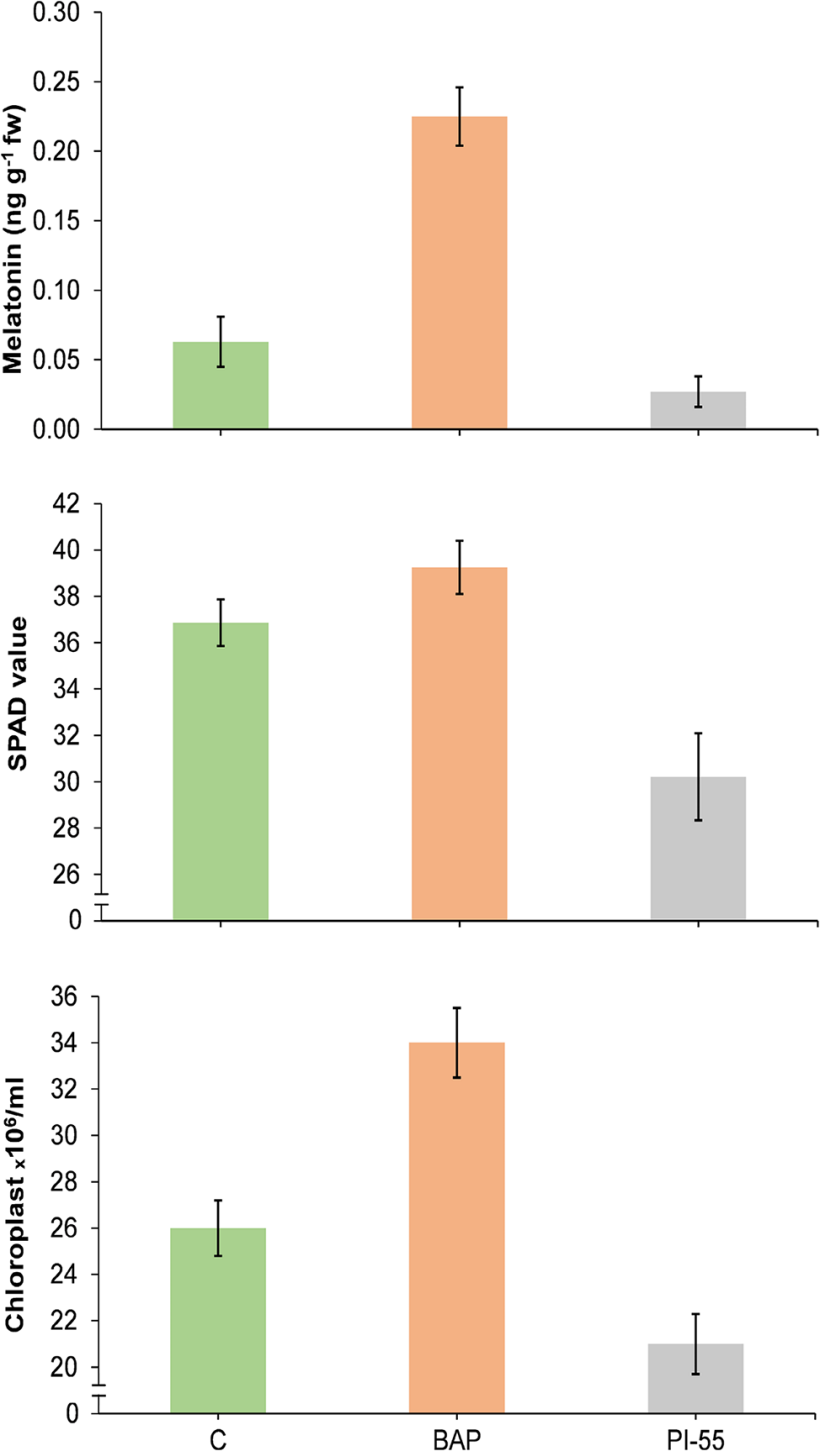
Evaluation of melatonin contents, SPAD value, and chloroplast numbers in leaves of the susceptible wheat genotype treated with BAP and PI-55. The values shown are means ± SE

The application of BAP significantly upregulated the expression of *CGA1* and *SNAT* (Fig. 8). In addition, it led to a significant increase in melatonin content, SPAD value, and chloroplast number (Fig. 9). In contrast, treatment with PI-55 downregulated *CGA1* and *SNAT* expression (Fig. 8) and caused a significant reduction in melatonin content, SPAD value, and chloroplast number compared to control plants (Fig. 9).

## Discussion

Melatonin unquestionably plays a multifaceted role in plants, particularly in enhancing stress tolerance. Its exogenous application induces dose-dependent effects on downstream responses. Higher doses of melatonin enhance antioxidant activity and reduce ROS accumulation, improving salinity tolerance in wheat [45]. In maize seedlings, exogenous melatonin has been shown to enhance drought tolerance by regulating growth, physiology, and gene expression [46]. In addition, under stress conditions, endogenous melatonin levels vary across different tissues (such as leaves, roots, or stems), influencing the ability of each tissue to tolerate stress [13, 28]. Tissues with higher melatonin concentration levels are better equipped to cope with stress, whereas those with lower levels tend to be less resilient [47]. This is due to melatonin’s protective role, with its concentration directly influencing the degree of stress tolerance in each plant tissue. Similarly to other phytohormones, the regulation of melatonin metabolism is a critical factor in fine-tuning its endogenous levels and, thereby, its function. However, the underlying mechanisms controlling melatonin biosynthesis, especially under stress conditions in wheat, remain largely unexplored. Studying gene expression regulation within key biological pathways is essential for understanding their functional roles and identifying targeted strategies to enhance plant stress tolerance. To achieve this, we conducted an integrated study combining computational and experimental methodologies to explore key aspects of the melatonin biosynthesis pathway in wheat.

### Melatonin mitigates drought stress in wheat

Core genes involved in the melatonin biosynthesis pathway of plants, including *SNAT*, have been identified and studied in various plant species. Furthermore, *SNAT* expression levels fluctuate in response to different abiotic stresses, influencing melatonin production and stress tolerance. The *SNAT* gene in *Malus zumi* is induced by drought, and transgenic Arabidopsis ectopically expressing *MzSNAT5* exhibits increased melatonin levels and enhanced drought tolerance [48]. In grape (*Vitis vinifera*), *SNAT1* is a salt-inducible gene highly expressed in leaves, and its overexpression in Arabidopsis significantly enhances salt tolerance by increasing melatonin accumulation and reducing oxidative damage [49]. *SNAT3D* in cotton is functionally involved in melatonin synthesis, antioxidant enzyme activity, and Ca^2+^ content under salinity stress [50]. Similarly, *SNAT1* and *SNAT2* in *Hypericum perforatum* are highly expressed in the leaves in response to salinity and drought. Overexpression of *HpSNAT1* and *HpSNAT2* in Arabidopsis and *H. perforatum* resulted in significantly increased melatonin levels, and *SNAT* overexpressed in Arabidopsis plants resulted in enhanced root growth and improved ROS scavenging ability [51]. Consistent with these findings, our results revealed significant differences in *SNAT* expression and melatonin content between the two contrasting wheat genotypes. The drought-tolerant genotype Sirvan exhibited higher *SNAT* expression and melatonin levels at all time points measured after drought stress compared to the susceptible genotype Moghan3. Moreover, the physiochemical assays revealed different drought tolerance phenomena between the genotypes, where Sirvan seems to be equipped with strengthened stress tolerance traits. Accordingly, water response and photosynthesis capacity in Sirvan were considerably higher than in Moghan3 during drought. Greater WUE and enhanced photosynthetic capacity are key indicators of crop drought tolerance [4].

Previous studies have shown that melatonin alleviates drought-induced growth inhibition and enhances photosynthetic capacity in maize and wheat seedlings [13, 52]. We observed a strong positive correlation between melatonin content and photosynthetic parameters, including SPAD value, *A*_N_, and WUEi. Additionally, PCA revealed a closer clustering of melatonin content, SPAD value, *A*_N_, and WUEi to the tolerant genotype, indicating higher levels of these characteristics in the tolerant genotype compared to the susceptible genotype. These findings suggest that elevated melatonin levels improve wheat photosynthetic efficiency under drought conditions through three key mechanisms. First, melatonin protects the chloroplast structure from ROS, as evidenced by the relatively high SPAD values observed in Sirvan during drought stress. This genotype also exhibited lower levels of H_2_O_2_ and MDA, suggesting an improved antioxidant status compared to Moghan3. Melatonin acts both as an antioxidant and activator of the plant’s antioxidant system, effectively eliminating scavenging ROS and reducing stress-induced damage [13, 53, 54]. Notably, chloroplasts are significant sources of ROS and are particularly vulnerable to oxidative stress compared to other cellular compartments [55–57]. Therefore, elevated melatonin levels in wheat leaves may protect the photosynthetic apparatus from the deleterious effects of drought. Similar protective effects have been reported in tomato, alfalfa, and maize under drought stress [58–60]. Second, the observed WUEi levels, which reflect the water content, indicate enhanced water potential and cell turgor in the drought-tolerant wheat genotype. This mechanism can enhance stomatal opening and conductance [61, 62]. In addition, melatonin may regulate the expression of genes involved in ABA biosynthesis, potentially increasing stomatal conductance, promoting water and CO_2_ movement, and ultimately enhancing photosynthesis [63, 64]. Melatonin also exerts regulatory control over the carbon fixation pathway, as evidenced by its upregulation of the RUBISCO (ribulose bisphosphate carboxylase) enzyme, thereby restoring photosynthetic performance under drought stress [65]. Moreover, increased expression of melatonin biosynthesis genes in Arabidopsis and *Phaseolus vulgaris* L. has been associated with elevated melatonin levels in chloroplasts, which enhances salt and high light tolerance through improved photosynthetic activity [66, 67]. Our results revealed a significantly higher *A*_N_ in Sirvan compared to Moghan3, indicating more efficient photosynthesis during drought stress. Part of this advantage can be attributed to the preservation of chloroplast structure and the regulation of stomatal conductance, as mentioned above. Another possible contributing factor is the potential effect of melatonin on RUBISCO in wheat, though this hypothesis requires additional investigation. The upregulation of *SNAT* gene expression, especially in the tolerant genotype, and the subsequent increase in melatonin are pivotal in alleviating drought stress in wheat. Therefore, *SNAT* emerges as a promising candidate for further research aimed at enhancing stress tolerance.

### Cytokinin signalling pathway and melatonin biosynthesis: the *CGA1-SNAT* module

In this study, we demonstrated that *SNAT* expression levels in wheat varied both within and between different genotypes under drought conditions, suggesting the presence of distinct regulatory mechanisms. We aimed to identify some of these mechanisms. Bioinformatics analysis revealed that *SNAT* expression differs across various tissue types and that a diverse set of TFs are likely involved in its regulation. These findings highlight the gene’s multifunctional roles in plants, particularly in wheat. Among the identified TFs potentially regulating *SNAT*, *CGA1* (also known as *GATA22*) was recognized as the most reliable predicted regulatory TF. It was positioned as a central gene in the derived gene regulatory network associated with *SNAT*. *CGA1* is a member of the GATA TF family, and these TFs are characterized by a conserved GATA motif, a DNA-binding domain that recognizes the consensus sequence 5’-GATA-3’ [68]. *CGA1* plays a crucial role in the cytokinin signalling pathway, which regulates various plant processes, including cell proliferation, differentiation, and responses to environmental stimuli [69, 70]. Based on our bioinformatics analysis and the findings related to *CGA1*, we hypothesize that there is a connection between cytokinin signalling and melatonin biosynthesis in wheat. To explore this, we first measured cytokinin concentrations in different wheat genotypes under drought conditions. Interestingly, under all experimental conditions, including non-stress conditions, cytokinin concentration in the tolerant genotype Sirvan was higher than that in the susceptible genotype Moghan3. Previous studies have demonstrated that higher levels of endogenous cytokinin enhance plant tolerance to diverse abiotic stresses, such as salinity, heat, and drought, mainly by regulating plant growth and stimulating the antioxidant system to remove ROS [71–73]. In wheat, cytokinin delayed the senescence of leaves and increased grain yield under water deficit stress conditions [74]. The activity of *CGA1* is essential for the precise regulation of cytokinin signalling and its expression is tightly controlled by cytokinin levels [75]. In response to elevated cytokinin concentrations, *CGA1* expression is upregulated, leading to the activation or repression of downstream target genes [69, 76]. This dynamic regulation allows plants to rapidly adjust their growth and development in response to changes in cytokinin levels, which may arise from developmental cues or environmental factors.

**Fig. 10:**
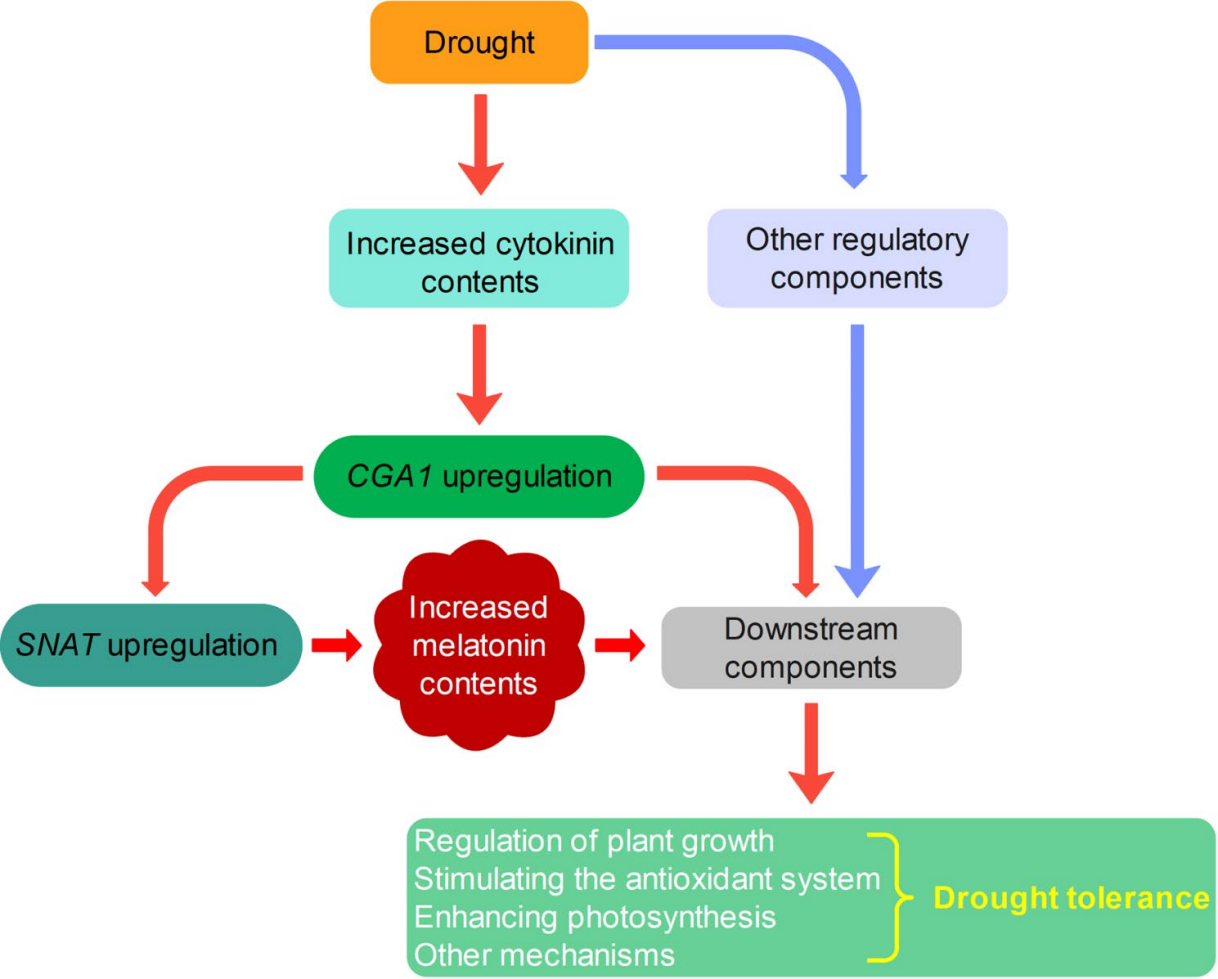
The postulated drought response pathways, including the *CGA1-SNAT* regulatory module and melatonin biosynthesis in wheat

Consistent with these findings, our results revealed that increased *CGA1* expression is positively correlated with elevated cytokinin concentrations in wheat leaves. To further support this hypothesis, we investigated the effects of exogenous cytokinin (BAP) and a cytokinin inhibitor (PI-55) on the expression levels of *CGA1* and melatonin contents in a susceptible wheat genotype. The results indicated that cytokinin can considerably upregulate *CGA1* expression and melatonin levels. While no previous studies have specifically demonstrated the impact of cytokinin concentration on *CGA1* expression in wheat, genome‑wide identification and characterization of GATA family genes in wheat has shown that *GATA* genes, including *CGA1*, are differentially expressed across various wheat tissues and under different abiotic stresses [77]. Moreover, BAP treatment significantly increased SPAD value and chloroplast number in leaves from Moghan3, suggesting an enhanced photosynthesis capacity, which is critical to stress tolerance. In rice, constitutive *CGA1* overexpression increases chloroplast biogenesis and starch production and results in delayed senescence and reduced grain filling by modulating essential nucleus-encoded, chloroplast-localized genes [78]. In addition, it has been reported that *CGA1* is involved in other pathways and modulated by light, nitrogen, and GA [44, 78]. Therefore, while its primary function is in cytokinin signalling, the interaction between the cytokinin pathway and other regulatory and functional pathways, especially the regulation of *SNAT* expression and melatonin biosynthesis pathway, implicates *CGA1* in broader stress adaptation mechanisms. This postulation is illustrated in Fig. 10.

## Conclusion

Our study revealed a strong association between melatonin variation and drought tolerance in wheat. Specifically, wheat genotypes with relatively higher melatonin levels exhibited significantly improved photosynthetic activity and antioxidant properties under drought conditions, indicating enhanced tolerance. Furthermore, our findings suggest that the regulation of *SNAT* gene expression by the *CGA1* TF is essential for conferring enhanced drought tolerance in wheat. The identified regulatory module, *CGA1-SNAT*, integrates the cytokinin signalling pathway with melatonin biosynthesis, presenting a promising direction for further research into hormone‒hormone interactions in wheat’s response to abiotic stress. Further functional characterization is essential for a comprehensive understanding of the precise roles and mechanisms of the *CGA1-SNAT* module.

## Electronic supplementary material

Below is the link to the electronic supplementary material.

Supplementary Material 1:Table S1 The list of primers used for expression analysis of wheat *SNAT* and *CGA1* genes.

Supplementary Material 2: Table S2 List of transcription factors (TFs) from 26 different families that may regulate the expression of the SNAT gene in wheat.

## Data Availability

Data is provided within the manuscript or supplementary information files.
