# Supplementary material for: The CGA1-SNAT regulatory module potentially contributes to cytokinin-mediated melatonin biosynthesis and drought tolerance in wheat
Source: BMC Plant Biol. 2025 Mar 7;25:296. doi: 10.1186/s12870-025-06313-3 (PMC11887191; doi:10.1186/s12870-025-06313-3)
Supplement: Supplementary file 1 — Supplementary Material 1:Table S1 The list of primers used for expression analysis of wheat SNAT and CGA1 genes. [file 12870_2025_6313_MOESM1_ESM.docx]

**Table S1**

The list of primers used for expression analysis of wheat *SNAT* and *CGA1* genes

| **Gene** | **Primer** | **Amplicon length on cDNA (bp)** |
| --- | --- | --- |
| ***SNAT*** | F 5^′^- AGGATGGCCACGCAGACCTC-3^′^ | 170 |
|  | R 5^′^-GTTGCTTCCTCTCTTCTCCCTC-3^′^ |  |
| ***CGA1*** | F 5^′^- AAGTCTCTTTGCAACGCATGCG-3^′^  R 5^′^-GCCTTCACTGCTTGTGATGCG-3^′^ | 164 |
| ***Actin*** | F 5^′^-TCAGAAAGGTTCAGGTGCC-3^′^ | 150 |
|  | R 5^′^-TCCACTGAGAACAACATTACC-3^′^ |  |
